# Gender minorities in breast cancer – Clinical trials enrollment disparities: Focus on male, transgender and gender diverse patients

**DOI:** 10.1016/j.breast.2024.103713

**Published:** 2024-03-09

**Authors:** Federica Miglietta, Letizia Pontolillo, Carmine De Angelis, Roberta Caputo, Monica Marino, Emilio Bria, Rossana Di Rienzo, Annarita Verrazzo, Carlo Buonerba, Giampaolo Tortora, Giuseppe Di Lorenzo, Lucia Del Mastro, Mario Giuliano, Filippo Montemurro, Fabio Puglisi, Valentina Guarneri, Michelino De Laurentiis, Luca Scafuri, Grazia Arpino

**Affiliations:** a Department of Surgery, Oncology and Gastroenterology, University of Padova, Padova, Italy; b Oncology 2 Unit, Istituto Oncologico Veneto - IOV IRCCS, Padova, Italy; c UOC Oncologia Medica, Comprehensive Cancer Center, Fondazione Policlinico Universitario Agostino Gemelli IRCCS, Rome, Italy; d Medical Oncology, Department of Traslational Medicine and Surgery, Università Cattolica del Sacro Cuore, Rome, Italy; e Department of Clinical Medicine and Surgery, University Federico II, Naples, Italy; f Department of Breast and Thoracic Oncology, Division of Breast Medical Oncology, Istituto di Ricovero e Cura a Carattere Scientifico (IRCCS) Pascale, Naples, Italy; g UOSD Oncologia Toraco-Polmonare, Comprehensive Cancer Center, Fondazione Policlinico Universitario Agostino Gemelli IRCCS, Rome, Italy; h Oncology Unit, Hospital “Andrea Tortora”, ASL Salerno, Pagani, Italy; i Associazione O.R.A. ETS – Oncology Research Assistance, Salerno, Italy; j UO Clinica di Oncologia Medica, IRCCS Ospedale Policlinico San Martino, Genoa, Italy; k Department of Internal Medicine and Medical Specialities, School of Medicine, University of Genoa, Genoa, Italy; l Breast Unit, Candiolo Cancer Institute, FPO-IRCCS, Candiolo, Italy; m Department of Medicine, University of Udine, Udine, Italy; n Department of Medical Oncology, Centro di Riferimento Oncologico di Aviano (CRO), IRCCS, Aviano, Italy

**Keywords:** Breast cancer, Gender minorities, Male, Transgender and gender diverse people, Gender diversity

## Abstract

**Background:**

The last years have seen unprecedented improvement in breast cancer (BC) survival rates. However, this entirely apply to female BC patients, since gender minorities (male, transgender/gender-diverse) are neglected in BC phase III registration clinical trials.

**Methods:**

We conducted a scoping review of phase III clinical trials of agents with a current positioning within the therapeutic algorithms of BC.

**Results:**

We selected 51 phase III trials. Men enrollment was allowed in 35.3% of trials. In none of the trial inclusion/exclusion criteria referred to transgender/gender-diverse people. A numerical higher rate of enrolled men was observed in the contemporary as compared to historical group.

We found a statistically significant association between the drug class and the possibility of including men: 100%, 80%, 50%, 33.3%, 25%, 10% and 9.1% of trials testing ICI/PARP-i, ADCs, PI3K/AKT/mTOR-i, anti-HER2 therapy, CDK4/6-i, ET alone, and CT alone.

Overall, 77409 patients were enrolled, including 112 men (0.2%). None of the trial reported transgender/gender-diverse people proportion. Studies investigating PARP-i were significantly associated with the highest rate of enrolled men (1.42%), while the lowest rates were observed for trials of CT (0.13%), ET alone (0.10%), and CDK 4/6-I (0.08%), p < 0.001.

**Conclusions:**

We confirmed that gender minorities are severely underrepresented among BC registration trials. We observed a lower rate of men in trials envisaging endocrine manipulation or in less contemporary trials. This work sought to urge the scientific community to increase the awareness level towards the issue of gender minorities and to endorse more inclusive criteria in clinical trials.

## Highlights

• Male are severely under-represented among registration trials of breast cancer.
• A lower rate of men was observed in trials envisaging endocrine manipulation or in less contemporary trials.
• Transgender and gender diverse people are neglected in the experimental scenario of breast cancer.
• These findings solicit the adoption of more inclusive criteria for enrollment in registration clinical trials.

## Graphical abstract

**Figure:**
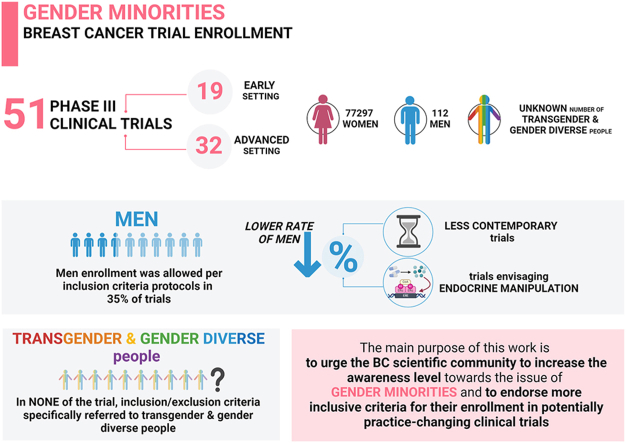

## 1 Introduction

Breast cancer (BC) represents the most frequently diagnosed solid cancer and the second leading cause of cancer-related death among women world-wide, covering, respectively, 31% and 15% of the estimated new cancer cases and deaths [1]. In the last decade we have witnessed tremendous improvements in terms of diagnostic capabilities and therapeutic opportunities leading to unprecedented BC-associated survival rates [2]. However, these breakthroughs almost entirely apply to cisgender female BC (women whose gender identity aligns with society's expectations based on their sex assigned at birth [3]), since gender minorities, namely male and transgender (people whose gender identity and/or gender expression does not align with society's expectations based on their sex assigned at birth [3]) and gender diverse people (individuals whose gender identity and/or gender expression deviates from social expectations; it includes terms as non-binary, gender-fluid, gender-queer, gender-neutral, a-gender, X-gender and many others [3,4]) are usually neglected in the experimental and clinical scenario of BC. In this context, despite an increasing awareness of cancer disparities in terms of gender identity, so far, the efforts and resources put at the service of this burning issue have been limited.

Male BC covers approximately 1% of all BC diagnoses thus representing a rare entity [5]. Notably, male BC is characterized by distinct clinicopathologic features, often reflecting and/or driving more unfavorable clinical outcome as compared to female BC [[6], [7], [8], [9]]. Accordingly, there is an urgent need to generate more solid data regarding the clinical value of available antitumor agents specifically in this patient population.

Regarding BC in transgender and gender diverse patients, available evidence is too scattered to formulate an estimation of BC risk, thus precluding the possibility to define the scale of the problem. Available data suggest that subjects identifying as non-cisgender may vary from approximately 0.1% to 6^10,11^%, covering up to 25% of the lesbian, gay, bisexual, transgender, queer, intersex and asexual (LGBTQIA+) community. However it is reasonable to speculate an underestimation of this phenomenon due to underreporting [[10], [11], [12], [13]]. In this scenario, there is a strong rational to assume that assimilating transgender and gender diverse subjects to the male/female cisgender dichotomy in terms of BC management might represent an unacceptable over-simplification. Indeed, it has been reported that subjects belonging to gender minorities may have a different attitude and propensity to screening and early detection measures potentially affecting BC clinical presentation at diagnosis. In addition, they may experience a higher burden of psycho-social distress, often correlated to a greater level of perceived discrimination, which may result, among other, in restricted treatment compliance and adherence. Moreover, type and depth of medical/surgical transition in transgender subjects may have a profound – albeit currently not estimable – impact on the considerations that can be made from a therapeutic point of view.

Overall, available evidence outlines a no longer acceptable gap between gender minorities specific clinical needs, clinical practice and the desirable production of evidence-based clinical practice guidelines.

In the present work we sought to review and analyze available evidence regarding the inclusion of gender minority subpopulations (men, transgender and gender diverse people) in phase III clinical trials leading to regulatory positioning of the most contemporary therapeutic treatment strategies across all BC subtypes and disease settings. The main purpose is to urge the BC scientific community to increase the awareness level towards this issue and endorse more inclusive criteria for the enrollment in potentially practice-changing clinical trials.

## 2 Methods

We conducted a scoping review of phase III clinical trials leading – from year to year - to registration of agents with a current positioning within the therapeutic algorithms of early and advanced BC. We matched FDA/EMA labels with ESMO/NCCN guidelines (at June 2023) to identify phase III clinical trials leading to registration of drugs with a current clinical positioning. Per each identified trial, information about year of publication (at time of first data presentation), disease setting, molecular subtype, class of drug, drug/regimen tested, inclusion of men and transgender [3] and gender diverse [3] people, was collected. Class of drug was categorized as follow: I) chemotherapy (CT) strategies; II) anti-HER2 treatments (excluding antibody drug conjugates [ADC]; III) endocrine therapy alone (ET); IV) cyclin-dependent kinase 4 and 6 inhibitors (CDK 4/6i); V) ADCs; VI) immunotherapy (ICI); VII) PARP-inhibitors; VIII) PI3K/AKT/mTOR inhibitors.

Statistical analyses were performed using IBM software SPSS v.24 (RRID:SCR_002865). Descriptive statistics were performed to analyze inter-studies differences in terms gender minorities’ accrual. Mean and inter-range values were computed for continuous variables. The Kolmogorov-Smirnov non parametric test was applied to assess the normal distribution of continuous variables and the Student-T test was applied to compare mean values of normally distributed variables. The Chi-squared test (χ2) was applied to make comparisons of categorical variables across subgroups. The statistical significance was set for p values < 0.05.

## 3 Results

### 3.1 Clinical trial characteristics and gender minorities enrolled

Overall, 51 phase III clinical trials published from 2000 to 2022 were identified and included in our review. Detailed characteristics of the studies included in the present analysis are reported in Table I.

**Table 1:** Characteristics of the studies included in the present analysis.

Trial	Year	Setting	Subtype	Class of Drug	Drug/regimen tested	Men enrollment per inclusion criteria	N pts enrolled	N men enrolled
Nabholtz et al. **North American Multicenter Randomized Trial** [14]	2000	Metastatic	HR+/HER2-	ET	Anastrozole *vs.*Tamoxifen	No	353	NA
Mouridsen et al. **Phase III study of the International Letrozole Breast Cancer Group** [15]	2001	Metastatic	HR+/HER-2-	ET	Letrozole *vs.* Tamoxifen	No	907	NA
Slamon et al. **Use of Chemotherapy plus a Monoclonal Antibody against HER2 for Metastatic Breast Cancer That Overexpresses HER2** [16]	2001	Metastatic	HER-2+	Anti-HER2 (no ADC)	CT +/H	No	469	NA
Baum et al. **ATAC** [17]	2002	Early/Adjuvant	HR+/HER2-HER2+	ET	Anastrozole vs. Tamoxifen	No	9366	NA
Bear et al. **NSABP** **PROTOCOL B-27** [18]	2003	Early	HR+/HER-2-TNBC	CT	AC-T-surgery *vs*. AC-Surgery *vs.* AC-Surgery-T	No	2411	NA
Citron et al. **Intergroup Trial C9741/Cancer and Leukemia Group B Trial 9741** [19]	2003	Early/adjuvant	HR+/HER-2-TNBC	CT	CT dose dense *vs.* conventionally scheduled CT	No	2005	NA
Piccart-Gebhart **HERA** [20]	2005	Early/adjuvant	HER-2+	Anti-HER2 (no ADC)	H 1 y *vs*. 2y *vs*. Observation	No	5081	NA
Cataliotti et al. **PROACT** [21]	2006	Early/neoadjuvant	HR+/HER-2-	ET	Anastrozole *vs.* tamoxifen	No	451	NA
Jones et al. **US Oncology Research Trial 9735** [22]	2006	Early/Adjuvant	HR+/HER-2-TNBC	CT	AC *vs.* TC	No	1016	NA
Paridaens et al. **Phase III Study Comparing Exemestane With Tamoxifen As First-Line Hormonal Treatment of Metastatic Breast Cancer in Postmenopausal Women: The European Organisation for Research and Treatment of Cancer Breast Cancer Cooperative Group** [23]	2008	Metastatic	HR+/HER-2-TNBC	ET	Exemestane *vs.* Tamoxifen	No	371	NA
Gianni et al. **NOAH** [24]	2010	Early/Neoadjuvant	HER-2+	Anti-HER2 (no ADC)	CT ± H	No	235	NA
Leo et al. **CONFIRM** [25]	2010	Metastatic	HR+/HER-2-	ET	Fulvestrant 250 mg *vs.* Fulvestrant 500 mg	No	736	NA
Cortés et al. **EMBRACE** [26]	2011	Metastatic	HR+/HER-2-HER-2+ TNBC	CT	Eribulin *vs.* TPC	No	762	NA
Slamon et al. **Adjuvant H in HER2-Positive Breast Cancer** [27]	2011	Early/adjuvant	HER-2+	Anti-HER2 (no ADC)	AC -T + H *vs.* AC-T *vs.* T + Carbolatin + H	No	3222	NA
Baselga et al. **BOLERO-2** [28]	2012	Metastatic	HR+/HER2-	PI3K/AKT/mTORi	Everolimus + exemestane *vs.* Exemestane + Placebo	No	724	NA
Davies et al. **ATLAS** [29]	2012	Early/Adjuvant	HR+/HER2-	ET	Tamoxifen	No	12894	NA
Swain et al. **CLEOPATRA** [30]	2012	Metastatic	HER2+	Anti-HER2 (no ADC)	T + H + P vs. T + H + Placebo	Yes	808	2
Verma et al. **EMILIA** [31]	2012	Metastatic	HER-2+	ADC	TDM-1 *vs.* lapatinib + Capecitabine	Yes	991	5
Iwata et al. **A randomized, double-blind, controlled study of exemestane versus anastrozole for the first-line treatment of postmenopausal Japanese women with hormone-receptor-positive advanced breast cancer.** [32] .	2013	Metastatic	HR+/HER2-HER2+	ET	Exemestane *vs.* Anastrozole	No	298	NA
Nitz et al. **WSG-AGO epiribicine and cyclophosphamide (EC)-Doc** [33]	2014	Early/Adjuvant	HR+/HER-2-HER-2+ TNBC	CT	EC *vs.* FEC	No	2012	NA
Del Mastro et al. **GIM-2** [34]	2015	Early/adjuvant	HR+/HER-2-HER-2+ TNBC	CT	CT dose dense *vs.* CT standard interval	No	2091	NA
Kaufman et al. **E7389-G000**–**301** [35]	2015	Metastatic	HR+/HER-2-HER-2+ TNBC	CT	Eribulin *vs.* Capeciitabine	No	1102	NA
Pivot et al. **CEREBEL** [36]	2015	Metastatic	HER2+	Anti-HER2 (no ADC)	Lapatinib-Capecitabine *vs.* H-Capecitabine	No	540	NA
Rugo et al. **CALGB 40502/NCCTG N063H (Alliance)** [37]	2015	Metastatic	HR+/HER2-HER2+ TNBC	CT	Paclitaxel vs. Nab-Paclitaxel	Yes	799	11
Blum et al. **The ABC Trials:** **USOR 06**–**090, NSABP B-46-I/USOR 07132, and NSABP B-49** [38]	2016	Early/Adjuvant	HR+/HER2-TNBC	CT	TC *vs.* AC	No	4242	NA
Chan et al. **EXTENET** [39]	2016	Early/Adjuvant	HER-2+	Anti-HER2 (no ADC)	Neratinib *vs.* Placebo	No	2840	NA
Cristofanilli et al. **PALOMA 3** [40]	2016	Metastatic	HR+/HER-2-	CDK4/6i	Fulvestrant + Palbociclib/placebo	No	521	NA
Finn et al. **PALOMA-2** [41]	2016	Metastatic	HR+/HER2-	CDK4/6i	Palbociclib + Letrozole *vs.*Placebo + Letrozole	No	666	NA
Hortobagyi et al. **MONALEESA-2** [42]	2016	Metastatic	HR+/HER-2-	CDK4/6i	Letrozole + Ribociclib/Placebo	No	668	NA
Robertson et al. **FALCON** [43]	2016	Metastatic	HR+/HER-2-	ET	Fulvestrant *vs.* Anastrozole	No	462	NA
Goetz et al. **MONARCH 3** [44]	2017	Metastatic	HR+/HER-2-	CDK4/6i	AI + Abemaciclib/Placebo	No	493	NA
Robson et al. **OlympiAD** [45]	2017	Metastatic	HR+/HER-2-TNBC	PARP-i	Olaparib vs. TPC	Yes	302	7
Sledge et al. **MONARCH-2** [46]	2017	Metastatic	HR+/HER-2-	CDK4/6i	Fulvestrant + Abemaciclib/placebo	No	669	NA
Von Minckwitz et al. **APHINITY** [47]	2017	Early/Adjuvant	HER2+	Anti-HER2 (no ADC)	CT + H + P *vs.* CT + H + Placebo	Yes	4805	11
Litton et al. **EMBRACA** [48]	2018	Metastatic	HR+/HER-2-TNBC	PARP-i	Talazoparib *vs.* CT	Yes	431	7
Loibl et al. **BRIGHTNESS** [49]	2018	Early/neoadjuvant	TNBC	CT	Paclitaxel ± Carboplatin/Placebo -Veliparib/Placebo	No	634	NA
Schmid et al. **IMpassion130** [50]	2018	Metastatic	TNBC	ICI	Nab-paclitaxel ± Atezolizumab	Yes	902	4
Slamon et al. **MONALEESA-3** [51]	2018	Metastatic	HR+/HER-2-	CDK4/6i	Fulvestrant + Ribociclib/Placebo	Yes	726	0
Tripathy et al. **MONALEESA-7** [52]	2018	Metastatic	HR+/HER-2-	CDK4/6i	Tamoxifen or AI + LHRHa + Ribocilib/Placebo	No	672	NA
Tutt et al. **TNT** [53]	2018	Metastatic	TNBC	CT	Carboplatin *vs.* T	No	376	NA
Andrè et al. **SOLAR-1** [54]	2019	Metastatic	HER-2+	PI3K/AKT/mTORi	Fulvestrant + Alpelisib/Placebo	Yes	572	1
von Minckwitz et al. **KATHERINE** [55]	2019	Early/Adjuvant	HER-2+	ADC	TDM-1 *vs*. H	No	1486	NA
Cortes et al. **KEYNOTE-355** [56]	2020	Metastatic	TNBC	ICI	CT + Pembrolizumab/Placebo	Yes	847	0
Johnston et al. **MONARCH-E** [57]	2020	Early/Adjuvant	HR+/HER-2-	CDK4/6i	ET ± Abemaciclib	Yes	5637	36
Murthy et al. **HER2CLIMB** [58]	2020	Metastatic	HER-2+	Anti-HER2 (no ADC)	H + Capecitabine + Tucatinib/Placebo	Yes	612	5
Schmid et al. **KEYNOTE-522** [56]	2020	Early/neoadjuvant	TNBC	ICI	CT + Pembrolizumab/Placebo	Yes	1174	1
Bardia et al. **ASCENT** [59]	2021	Metastatic	TNBC	ADC	SG vs. CT	Yes	468	2
Tutt et al. **OLYMPIA** [60]	2021	Early/Adjuvant	HR+/HER-2-TNBC	PARP-i	Olaparib *vs.* Placebo	Yes	1836	6
Bidard et al. **EMERALD** [61]	2022	Metastatic	HR+/HER-2-	ET	Elacestrant *vs.* standard ET	Yes	705	7
Cortés et al. **DESTINY-Breast03** [62]	2022	Metastatic	HER2+	ADC	TDX-d *vs.* TDM-1	Yes	524	2
Rugo et al. **TROPICs-02** [63]	2022	Metastatic	HR+/HER-2-TNBC	ADC	Sacituzumab-Govitecan *vs.* TPC	Yes	543	5

Abbreviations: AC: doxorubicin and cyclophosphamide; AI: aromatase inhibitor; ADC: antibody drug conjugates; CT: Chemotherapy; EC: epirubicine and cyclophosphamide; ET: endocrine therapy; FEC: 5-fluorouracil, epidoxorubicin and cyclophosphamide; H: Trastuzumab; HR: hormone receptor positive; N= Number; P: Pertuzumab; T: Docetaxel; TC: Docetaxel and cyclophosphamide; TDM-1: Trastuzuamb-emtansine; TDX-d: Trastuzumab-deruxtecan; TNBC: triple negative breast cancer; TPC: treatment of physician's choice.

Thirty-two (62.7%) and 19 (37.2%) trials were conducted respectively in the metastatic and early (either neoadjuvant or adjuvant) setting.

When classified based on the subtype, the most represented was hormone receptor positive- HER2-negative (HR+/HER2-, n = 32, 62.7%), followed by HER2 positive (HER2+, n = 20, 39.2%) and triple negative BC (TNBC, n = 20, 39.2+%).

Based on the class of drug, 11 (21.6%) trials investigated CT based strategies, 10 (19.6%) ET, 9 (17.6%) anti-HER2 treatments (excluding ADC), 8 (15.7%) CDK4/6i, 5 (9.8%) ADC, 3 (5.9%) ICI, 3 (5.9%) PARP- inhibitors and 2 (3.9%) PI3K/AKT/mTOR inhibitors respectively.

Looking at the population of the trials included in the present analysis, overall, 77409 patients were enrolled: 77297 women (99.8%) and 112 men (0.2%). Only 18 (35.3%) of 51 trials allowed men to be enrolled in the study as per inclusion criteria.

Although none of the included trial referred to transgender and gender diverse patients as inclusion/exclusion criteria or mentioned the number enrolled, in one of them, concurrent hormone replacement therapies represented an exclusion criterion, while in another one prior estrogen and/or progesterone-containing hormone preparations for nononcologic purposes was allowed, as long as it was discontinued prior to registration. Due to the lacking of data regarding the transgender and gender diverse subpopulation, we excluded this subgroup from our analyses.

### 3.2 Evolution over time and according to disease setting

Based on the progressive expansion of the therapeutic armamentarium for BC patients, including new generation agents, we firstly evaluated the evolution of the inclusion of gender minorities in BC trials over time.

**Fig. 1:**
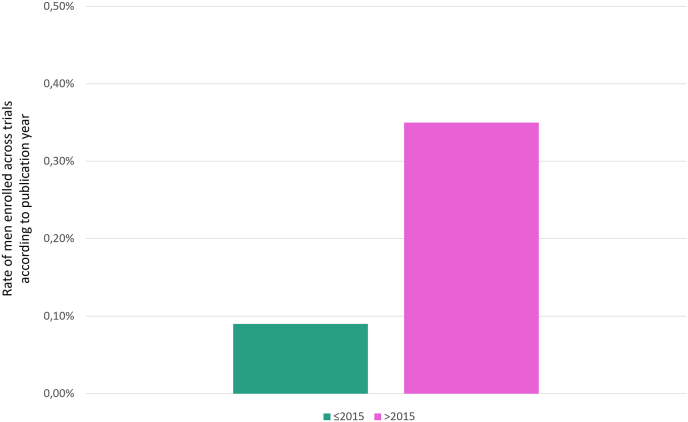
Rate of men enrolled across trials according to publication year (historical [≤2015] vs contemporary [>2015]).

**Fig. 2:**
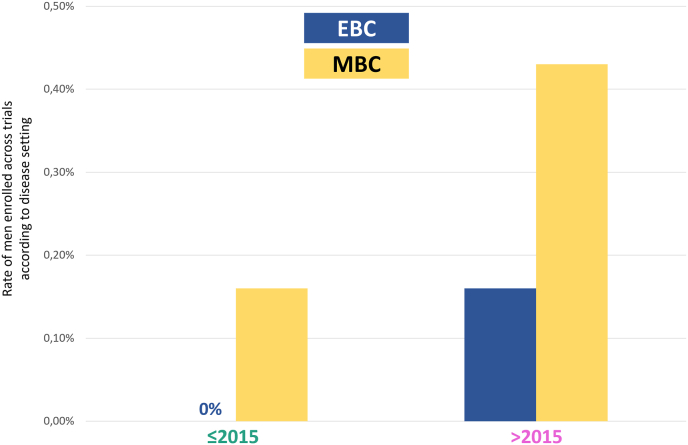
Rate of men enrolled across trials according to disease setting (early vs advanced), stratified per publication year (≤2015 vs > 2015) Abbreviations: EBC: early breast cancer; MBC: metastatic/advanced breast cancer.

Adopting the year 2015 as cutoff, we stratified trials into two groups: “contemporary” including trials published later than 2015 and “historical” including trials published earlier or in 2015. Overall, 7 (13.7%) and 46 (86.3%) trials were categorized as contemporary or historical, respectively. A numerical higher rate of enrolled men was observed in the contemporary group (0.35%) as compared to historical group (0.09%) (Fig. 1). Interestingly, most men enrolled in contemporary trials had metastatic breast cancer. In detail, considering the contemporary trials, the rate of enrolled men was 0.43% in the metastatic setting versus 0.16% in the early setting; conversely, in historical trials, the rate of men enrollment was 0.16% in the advanced setting with no men enrolled in the early setting (Fig. 2).

As mentioned above, only a minority of trials was conducted in patients with early-stage disease. We performed two different analyses to investigate how the disease setting impacted on the possibility of enrolling men per inclusion criteria and on the number of men actually enrolled.

No difference was showed in terms of men enrollment per inclusion criteria in the early versus advanced setting.

**Fig. 3:**
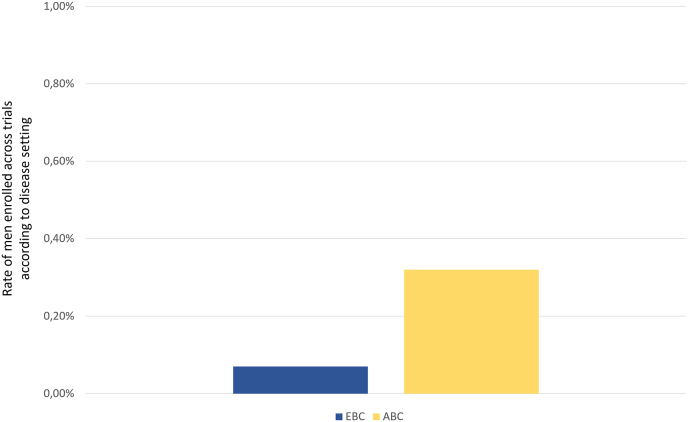
Rate of men enrolled across trials according to disease setting (early vs advanced) Abbreviations: EBC: early breast cancer; MBC: metastatic/advanced breast cancer.

When assessing the rate of men enrolled across trials according to the disease setting, a numerical higher rate of men was enrolled across trials conducted in the advanced setting (0.32%) as compared to the early setting (0.07%), Fig. 3.

### 3.3 Enrollment according to drug class

Finally, we investigated the association between the drug class and both men enrollment allowance per inclusion criteria and the number of men effectively enrolled.

**Fig. 4:**
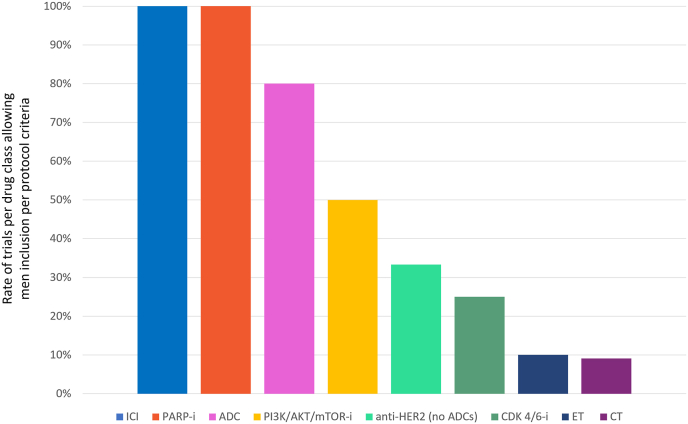
Rate of trials per drug class allowing men inclusion per protocol criteria Abbreviations: ICI: immune checkpoint inhibitors; PARP-i: Poly (ADP-ribose) polymerase inhibitors; ADC: antibody drug conjugate; PI3K/AKT/mTOR-i: Phosphoinositide 3-kinases/AKT/mammalian target of rapamycin inhibitors; CDK 4/6-i: cyclin-dependent kinase 4 and 6 inhibitors; ET: endocrine therapy; CT: chemotherapy.

Regarding inclusion criteria, men were allowed in 3 (100%), 4 (80%), 1 (50%), 3 (33.3%), 2 (25%), 1 (10%) and 1 (9.1%) trials testing ICI or PARP-I inhibitors, ADCs, PI3K/AKT/mTOR inhibitors, anti-HER2 therapy (excluding ADC), CDK4/6 inhibitors, ET alone, and CT alone respectively with a statistically significant difference across the groups (p = 0.002) (Fig. 4).

**Fig. 5:**
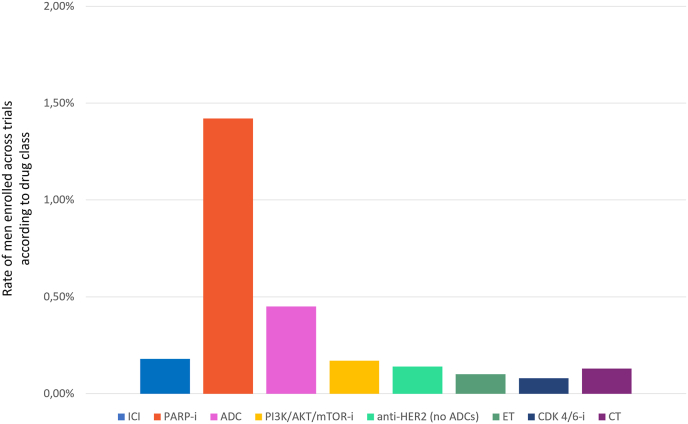
Rate of men enrolled across trials according to drug class Abbreviations: ICI: immune checkpoint inhibitors; PARP-i: Poly (ADP-ribose) polymerase inhibitors; ADC: antibody drug conjugate; PI3K/AKT/mTOR-i: Phosphoinositide 3-kinases/AKT/mammalian target of rapamycin inhibitors; ET: endocrine therapy; CDK 4/6-i: cyclin-dependent kinase 4 and 6 inhibitors; CT: chemotherapy.

Among trials allowing men inclusion per protocol criteria, studies investigating PARP inhibitors were significantly associated with the highest rate of enrolled men (1.42%), followed by ADC (0.45%), ICI (0.18%), PI3K/AKT/mTOR inhibitors (0.17%), anti-HER2 therapy (excluding ADC, 0.14%), with the lowest rates observed for trials of CT (0.13%), ET alone (0.10%), and CDK 4/6 inhibitors (0.08%), p < 0.001 (Fig. 5).

## 4 Discussion

In the present work we conducted a scoping review with also descriptive statistics focusing on inclusion of gender minority subpopulations (men, transgender and gender diverse people) in phase III clinical trials leading to regulatory positioning of the most contemporary therapeutic treatment strategies in BC.

We found that approximately one third of trials allowed the inclusion of men per protocol criteria, and this was strictly related to the therapeutic class investigated. In particular, while all trials testing ICI and PARP-inhibitors allowed the inclusion of men, this proportion decreased to 50–80% in trials testing ADC and/or anti-HER2 treatments, dropping to 25% for CDK 4/6 inhibitors, with the lowest rates observed for trials of ET and CT alone. Consistently, although the rate of male patients that were actually included was generally extremely low across all trials, it was significantly higher within trials testing PARP-inhibitors, with rates <1% for all the other therapeutic strategies. Importantly, we also found that more contemporary trials, especially those conducted in the metastatic setting, were associated with higher rates of enrolled men as compared to more historical ones.

These observations arise several orders of considerations. Firstly, the suboptimal coverage of male BC minority that we have reported within BC pivotal trials outlining the current therapeutic landscape of BC, may be at least in part driven by the low expected accrual of men with BC. However, international drug agencies currently do not consider this aspect - when the only one to stand – as a sufficient reason for excluding men from pre-marketing clinical trials [64].

Secondly, it was not surprising to confirm [65] in a contemporary experimental environment that trials envisaging endocrine-based strategies (as part of the randomization process or mandated per protocol criteria) typically tended to exclude male patients, thus driving the lowest rate of men included over the total enrolled population. Possible underlying reasons may be represented by the paucity of solid data regarding the effectiveness and safety of endocrine therapy as well as the concerns regarding the adherence to this treatment strategy within the male BC subgroup. Indeed, it has been reported that male BC patients tend to experience more treatment-limiting symptoms related to hormonal therapy that the female counterpart [66,67]. Importantly, this aspect may have deep implications in terms of suboptimality of male BC management since this entity is substantially enriched for HR+/HER2-cases [7], thus making endocrine-based therapy the main backbone of treatment also for men, both in the early and advanced setting. Possible direct consequences of the insufficiency of randomized data addressing this gender minority are the mere extrapolation of data generated in fully-female BC trials for the definition of the treatment decision process in male patients or, even worse, the exclusion of men from the label of novel endocrine-based treatment strategies.

Accordingly, we also observed that trials testing targeted strategies not involved in endocrine manipulation, had typically less conservative inclusion criteria, with the possibility of enrolling men in all or most pivotal clinical trials. The observation that trials leading to the regulatory positioning of PARP-inhibitors had the highest proportion of enrolled men, well fit within this framework, being germline BRCA mutations the most relevant not endocrine-driven theragnostic biomarker for BC male patients [68]. On the other hand, it should be acknowledged that a not negligible proportion of non-endocrine trials excluded male BC patients without providing a scientific rational for this choice, with the unavoidable consequence of precluding BC male patients to get access to potentially effective treatment strategies also in the post-marketing scenario.

Although our work outlines a major and imperative room for improvement, the significant increase of the rate of men enrolled within pre-marketing trials over time appears reassuring. Although a possible explanation may be the enrichment of BC contemporary therapeutic experimental scenario with targeted non-endocrine-based strategies, this advancement may be also the driven by the progressive increase of the awareness level both from an investigator and a regulatory perspective regarding this burning issue.

Our results also show the complete lack of representation of gender minorities (transgender and gender diverse subjects) within BC pivotal clinical trials, thus making these subpopulations unacceptably neglected entities within the contemporary landscape. Available evidence suggests that transgender and gender diverse people's needs are under-estimated and under/mis-addressed in terms of primary and secondary prevention, and that sex and gender minorities typically experience disparities in the oncological care [13,69,70], driving poorer BC-related outcome. Our study perfectly fits within this framework, providing another important piece of the puzzle and arising important therapeutic considerations when dealing with gender minorities, including men and transgender and gender diverse people.

To conclude, the main purpose of this work was to urge the BC scientific community to increase the awareness level towards the issue of gender minorities and to endorse more inclusive criteria for their enrollment in potentially practice-changing clinical trials. On the same ground lie the ongoing effort within the scientific breast cancer oncology community, which is progressively recognizing the unique needs of gender-diverse people, providing recommendations [4] revolving around a multi/*trans*-disciplinary patient-centered approach, ultimately aimed at improving medical education on gender minorities special needs, promoting inclusive policies and ensuring equitable cancer care to cancer patients belonging to gender minorities. Indeed, the inclusion of transgender and gender diverse patients in cancer clinical trials may be promoted by: (i) implementing measures for the mitigation of socio-economic marginalization, (ii) tackling the preconceptions driven by previous discrimination experiences within the healthcare setting, (iii) avoiding presumptive language in the trial protocols, (iv) allowing gender affirming hormone therapy unless clearly contraindicated for scientifically documented safety reasons, (v) being inclusive towards HIV + patients with clinically acceptable CD4 count and compliant with antiretroviral treatments in the absence of scientifically documented interactions with the experimental regimen [4].

## Role of the funding source

The funders had no role in the design and conduct of the study; collection, management, analysis, and interpretation of the data; preparation, review, or approval of the manuscript; or decision to submit the manuscript for publication.

## CRediT authorship contribution statement

**Federica Miglietta:** Writing – review & editing, Writing – original draft, Visualization, Methodology, Investigation, Formal analysis, Data curation, Conceptualization. **Letizia Pontolillo:** Writing – review & editing, Writing – original draft, Methodology, Investigation, Formal analysis, Data curation, Conceptualization. **Carmine De Angelis:** Writing – review & editing, Investigation. **Roberta Caputo:** Writing – review & editing, Investigation. **Monica Marino:** Writing – review & editing, Investigation. **Emilio Bria:** Writing – review & editing, Investigation. **Rossana Di Rienzo:** Writing – review & editing, Investigation. **Annarita Verrazzo:** Writing – review & editing, Investigation. **Carlo Buonerba:** Writing – review & editing, Investigation. **Giampaolo Tortora:** Writing – review & editing, Investigation. **Giuseppe Di Lorenzo:** Writing – review & editing, Investigation. **Lucia Del Mastro:** Writing – review & editing, Investigation. **Mario Giuliano:** Writing – review & editing, Investigation. **Filippo Montemurro:** Writing – review & editing, Investigation. **Fabio Puglisi:** Writing – review & editing, Investigation. **Valentina Guarneri:** Writing – review & editing, Investigation. **Michelino De Laurentiis:** Writing – review & editing, Investigation. **Luca Scafuri:** Writing – review & editing, Writing – original draft, Investigation, Data curation. **Grazia Arpino:** Writing – review & editing, Supervision, Methodology, Investigation, Conceptualization.

## Declaration of competing interest

FM reports personal fees from Roche, Novartis, Pfizer, Seagen, Gilead, Astrazeneca, Lilly, Menarini all outside the submitted work (all the disclosed activities are outside the submitted work). CDA reports advisory role for Roche, Lilly, Novartis, Astrazeneca, Pfizer, Seagen, Daiichi-Sankyo, Gilead, and GSK and speaker honoraria from Roche, Lilly, Novartis, Pfizer, Seagen, GSK, GILEAD, and Daiichi-Sankyo; travel Grants from Gilead and research support (to the Institution) from Novartis, GILEAD, and Daiichi-Sankyo outside the submitted work (all the disclosed activities are outside the submitted work). RC reports fees for talk or consultation from Novartis, Lilly, MSD, Gilead, Roche, Pfizer, Veracyte, Seagen, Astra Zeneca, Daichii Sankyo, Menarini, Pierre-Fabre (all the disclosed activities are outside the submitted work). EB has received grants or contracts from Astra-Zeneca, Roche and honoraria for lectures from Merck-Sharp & Dome, Astra-Zeneca, Pfizer, Eli-Lilly, Bristol-Myers Squibb, Novartis, Takeda and Roche; he has been member of Data Safety Monitoring Board or Advisory Board of Merck-Sharp & Dome, Pfizer, Novartis, Bristol-Myers Squibb, Astra-Zeneca, and Roche (all the disclosed activities are outside the submitted work). AV reports payment or honoraria for lectures, presentations, speakers bureaus, manuscript writing or educational events: AndromedaE20, Oncotech; support for attending meetings and/or travel: Pierre Fabre, Lilly, Gilead, Seagen, Novartis, MSD, Menarini (all the disclosed activities are outside the submitted work). LDM reports institutional grants from Eli Lilly, Novartis, Roche, Daiichi Sankyo, Seagen, Astrazeneca, Gilead, Pierre Fabre; consulting fees: Eli Lilly, Gilead, Daiichi Sankyio; payment or honoraria for lectures, presentations, speakers bureaus: Roche, Novartis, Pfizer, Eli Lilly, Astrazeneca, MSD, Seagen, Gilead, Pierre Fabre, Eisai, Exact Sciences, Ipsen, GSK, Agendia, Stemline; support for attending meetings and/or travel: Roche, Pfizer, Eisai, Daiichi Sankyo, Astrazeneca, Gilead; Participation on a Data Safety Monitoring Board or Advisory Board: Novartis, Roche, Eli Lilly, Pfizer, Daiichi Sakyo, Exact Sciences, Gilead, Pierre Fabre, Eisai, Astrazeneca, Agendia, GSK, Seagen, Olema, MSD, Stemline Menarini (all the disclosed activities are outside the submitted work). FM reports consultancy fees from Roche, Novartis, AstraZeneca, Daiichy Sankyo, SeaGen, MSD, Eli Lilly, Pfizer, and Pierre Fabre, and Travel Grants from Roche and Astra Zeneca; from May 15th, 2023, FM is Roche employee (all the disclosed activities are outside the submitted work). VG reports personal fees for advisory board membership for AstraZeneca, Daiichi Sankyo, Eisai, Eli Lilly, Exact Sciences, Gilead, Merck Serono, MSD, Novartis, Pfizer, Olema Oncology, Pierre Fabre; personal fees as an invited speaker for AstraZeneca, Daiichi Sankyo, Eli Lilly, Exact Sciences, Gilead, GSK, Novartis, Roche and Zentiva; personal fees for expert testimony for Eli Lilly. All the remaining authors have no conflict of interest to report.
